# PACAP signaling is not involved in GTN- and levcromakalim-induced hypersensitivity in mouse models of migraine

**DOI:** 10.1186/s10194-022-01523-8

**Published:** 2022-12-05

**Authors:** Song Guo, Charlotte Ernstsen, Anders Hay-Schmidt, David Møbjerg Kristensen, Messoud Ashina, Jes Olesen, Sarah Louise Christensen

**Affiliations:** 1 Department of Neurology, Danish Headache Center, Research Institute, Copenhagen University Hospital-Rigshospitalet Glostrup, Nordstjernevej 42, Glostrup 2600 Copenhagen, Denmark; 2 Department of Odontology, Faculty of Health, Panum Institute, University of Copenhagen, Copenhagen, Denmark; 3 Department of Science and Environment, Roskilde University, Universitetsvej 1, Rosklide, Denmark; 4 Univ Rennes, INSERM, EHESP, Irset (Institut de recherche en santé, environnement et travail) - UMR_S 1085, Rennes, France; 5 Department of Neurology, Danish Headache Center, Human Migraine Research Unit, Copenhagen University Hospital Rigshospitalet-Glostrup, Copenhagen, Denmark

**Keywords:** PACAP, Levcromakalim, GTN, Migraine, Von Frey, Monoclonal antibodies

## Abstract

**Background:**

Calcitonin gene-related peptide (CGRP) antagonizing drugs represents the most important advance in migraine therapy for decades. However, these new drugs are only effective in 50–60% of patients. Recent studies have shown that the pituitary adenylate cyclase-activating peptide (PACAP38) pathway is independent from the CGRP signaling pathway. Here, we investigate PACAP38 signaling pathways in relation to glyceryl trinitrate (GTN), levcromakalim and sumatriptan.

**Methods:**

In vivo mouse models of PACAP38-, GTN-, and levcromakalim-induced migraine were applied using tactile sensitivity to von Frey filaments as measuring readout. Signaling pathways involved in the three models were dissected using PACAP-inhibiting antibodies (mAbs) and sumatriptan.

**Results:**

We showed that PACAP mAbs block PACAP38 induced hypersensitivity, but not via signaling pathways involved in GTN and levcromakalim. Also, sumatriptan has no effect on PACAP38-induced hypersensitivity relevant to migraine. This is the first study testing the effect of a PACAP-inhibiting drug on GTN- and levcromakalim-induced hypersensitivity.

**Conclusions:**

Based on the findings in our mouse model of migraine using migraine-inducing compounds and anti-migraine drugs, we suggest that PACAP acts via a distinct pathway. Using PACAP38 antagonism may be a novel therapeutic target of interest in a subgroup of migraine patients who do not respond to existing therapies.

## Introduction

Over the past 20 years two endogenous neuropeptides, calcitonin gene-related peptide (CGRP) and pituitary adenylate cyclase-activating peptide (PACAP) have been of increasing interest in migraine [1]. The development and introduction of drugs targeting CGRP or its receptors represent the most important advances in migraine therapy for decades [2]. However, CGRP signaling pathway targeted therapies are effective only in 50–60% of patients [3–6]. The mechanisms in 40% of the patients who do not respond to CGRP signaling pathway targeted therapies are still unknown, and here PACAP may be critically involved.. Like CGRP, PACAP induces migraine attacks when given intravenously to adults with migraine [7]. Targeting PACAP would therefore be a reasonable therapeutic approach for migraineurs. Accordingly, two monoclonal antibody (mAb) to PACAP are currently in clinical phase II development [8, 9]. However, more specific knowledge about its signaling pathway and mechanism of action in migraine is warranted.

Recently, rodent models of migraine showed that the PACAP38 pathway is independent from the CGRP signaling pathway [10, 11]. We also showed this both in knockout mice without functional CGRP receptors and by antibody neutralization of CGRP. Our findings are supported by data from another group using light aversion in mice as a surrogate for migraine-like photophobia to compare CGRP and PACAP38 [11]. These findings are important as PACAP38 is the only migraine trigger tested in our rodent model to bypass CGRP. Other known triggers including glyceryl trinitrate (GTN) and levcromakalim, act at least in part via the CGRP pathway [12–14]. How PACAP38 is involved in relation to other migraine triggers has only been investigated in few studies [15, 16]. More studies are needed to further clarify this aspect, as drugs against PACAP may provide a therapeutic option for patients who do not respond to anti-CGRP drugs. The aim of the present study was to investigate PACAP38 signaling pathways in relation to GTN, levcromakalim and sumatriptan.

## Materials and methods

### Experimental animals

We used in total 168 C57Bl/6JBomTaC wildtype (WT) mice (Taconic, Denmark) at 7–9 weeks of age with equal number of both sexes. The mice were acclimatized for 1 week before the beginning of experiments and weighed between 17 and 29 g. We observed no age-dependent differences. The mice were cared for under the same conditions as previously published [17]. Mice were housed in a temperature and light controlled room (lights on at 07:00 with a 12 h light/dark cycle) with food and water ad libitum in cages with shelters and nesting material for enrichment purposes. As a general health assessment, all mice were weighed on every test day. The experiments were conducted in accordance with ARRIVE guidelines and approved by the Danish Animal Experiments Inspectorate (ethical approval number 2017–15-0201–01,358).

### Experimental design and protocols

**Fig. 1:**
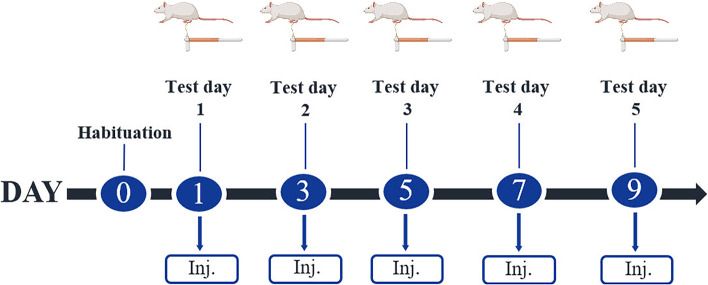
Design and experimental timeline of test paradigm for the injected trigger compounds. Following 1 day of habituation (day 0), five tests were done every other day over the course of 9 days. All compounds were injected intraperitoneally (i.p.) except for PACAP38 that was given subcutaneously (s.c.). Anti-PACAP antibodies (30 mg/kg) or IgG control (30 mg/kg) were injected once (day 0) 24 h prior to test day 1. Pre-treatment of sumatriptan (0.6 mg/kg) was given on every test day 20 min prior to the injection of PACAP38. On every test day, the basal threshold of cutaneous sensitivity was measured using Von Frey filaments prior to injections and the acute response after injections was measured after 1 h for PACAP38 and 2 h for GTN and levcromakalim

We used an in-house validated mechanistic mouse model of migraine using migraine-inducing compounds [12, 14, 17, 18] and evaluated effects by measurement of cutaneous sensitivity as previously described [19, 20]. The model uses compounds that trigger migraine (PACAP38, GTN or levcromakalim) injected into the mice followed by measurements using Von Frey filaments as a surrogate marker of migraine pain [20]. Table 1 provides an overview of the experiments and compounds applied. Group size was 12 in all experiments. Figure 1 provides the timeline of the complete study protocol. Mice of both sexes were tested every other day on 5 test days spanning over 9 days including injections followed by Von Frey testing. The GTN model has been thoroughly validated for its relevance to migraine and is commonly used [18, 20, 21] whereas the PACAP38 [10] and levcromakalim models are more novel [14, 22]. In four independent experiments, mice were either pre-treated with anti-PACAP mAbs or migraine-specific acute treatment (sumatriptan) followed by migraine provocation using the mouse model of migraine triggers. Each experiment was conducted separately using a new cohort of mice. Anti-PACAP antibodies or IgG control were injected once (day 0) 24 h prior to test day 1. Pre-treatment of sumatriptan was given on every test day 20 min prior to the injection of PACAP38. On every test day, the basal threshold of cutaneous sensitivity was measured prior to injections (that is 48 h after last injection), and the acute response after injections was measured after 1 h for PACAP38 and 2 h for GTN and levcromakalim. All tests were conducted in low-light conditions (20–30 lx) in the timeframe of 8:00–15:00 by a blinded experimenter.

**Table 1:** Overview of the experiments, test groups (*n* = 12), and compounds used

	**Pre-treatment compound**	** + **	**Test day compound**
**Experiment 1: Anti-PACAP mAbs + PACAP38**			
Group 1	IgG control (30 mg/kg)	+	Vehicle (saline)
Group 2	Anti-PACAP mAbs (30 mg/kg)	+	Vehicle (saline)
Group 3	IgG control (30 mg/kg)	+	PACAP38 (2 µg/kg)
Group 4	Anti-PACAP mAbs (30 mg/kg)	+	PACAP38 (2 µg/kg)
**Experiment 2: Anti-PACAP mAbs + GTN**			
Group 1	Anti-PACAP mAbs (30 mg/kg)	+	Vehicle (12.8% alcohol)
Group 2	IgG control (30 mg/kg)	+	GTN (10 mg/kg)
Group 3	Anti-PACAP mAbs (30 mg/kg)	+	GTN (10 mg/kg)
**Experiment 3: Anti-PACAP mAbs + Levcromakalim**			
Group 1	Anti-PACAP mAbs (30 mg/kg)	+	Vehicle (2% DMSO)
Group 2	IgG control (30 mg/kg)	+	Levcromakalim (1 mg/kg)
Group 3	Anti-PACAP mAbs (30 mg/kg)	+	Levcromakalim (1 mg/kg)
**Experiment 4: Sumatriptan + PACAP38**			
Group 1	Vehicle (saline)	+	Vehicle (saline)
Group 2	Vehicle (saline)	+	PACAP38 (2 µg/kg)
Group 3	Sumatriptan (0.6 mg/kg)	+	PACAP38 (2 µg/kg)

### Behavioral tests

#### Cutaneous sensitivity to tactile stimulation

We measured cutaneous sensitivity at pre-defined time points using von Frey filaments (0.008–2.0 g, not including 1.4 g, Ugo Basile, Italy) starting at 0.16 g on the left plantar area of hind paw. We used the up-down method [23] and calculated a withdrawal threshold stated in grams of 50% using a free online software program: https://bioapps.shinyapps.io/von_frey_app [24]. Cutaneous sensitivity testing of the plantar was performed with the mouse put in a clear plexiglas chamber with a mesh floor net (IITC Life Science). Before the first test day (day 0), mice were placed 45 min in the plexiglas chambers for habituation to experimental surroundings. Prior to each testing, mice were placed 30 min in test chambers for habituation [22].

#### Motor function (Rotarod)

General motor function was assessed using a rotarod (Rotarods Advanced, IITC Life Science Inc.) to show that the cutaneous sensitivity test using von Frey filaments was not biased by side effects of study drugs causing impaired motor function or sedation. The rotarod test was performed 24 h after injection of anti-PACAP mAb and right after the final von Frey test on the last study day (test day 9). Each mouse was given only one attempt and were placed on the rotarod with a start speed of 0 rpm which increased to 30 rpm with a ramp of 45 s and terminated after 150 s (max duration). The time spend on the rotarod was recorded. Midazolam (2 mg/kg, i.p.) or saline (i.p.) was used as positive/negative control injected 10 min prior to testing. Mice were randomized according to treatment groups when given midazolam.

### Test compounds

Compounds were injected intraperitoneally (i.p.) except for PACAP, which was administered subcutaneously (s.c.) with 25G needles (BD Microlance™ 3, BD, NJ, USA) in the lower right side of the abdomen. We injected a volume of 10 mL/kg (i.p.) except for PACAP38 (5 mL/kg, s.c.) and the antibodies (6 mL/kg, i.p.). The compounds were diluted in saline (Fresenius Kabi, Germany) unless otherwise specified. Compounds, vehicles, concentrations, and doses applied are summarized in Table 2.

**Table 2:** Overview of study compounds used in vivo (alphabetical order)

Compound	Provider	Mechanism of action	Dose, route of administration	Time of injection	Vehicle
GTN	Cambrex (Germany) via RegionH Pharmacy (Denmark)	Nitric oxide (NO) donor	10 mg/kg, i.p	2 h prior to test	12.2% alcohol in saline
Levcromakalim	Tocris, Bio-Techne Ltd (UK)	K_ATP_ channel opener	1 mg/kg, i.p	2 h prior to test	2% DMSO in saline
Anti-PACAP mAb	H. Lundbeck (Denmark)	Human anti-PACAP mAb	30 mg/kg, i.p	24 h before first test (pre-treatment)	IgG control mAb in vehicle provided by Lundbeck
Midazolam	Hameln Pharma GMBH (Germany) via RegionH Pharmacy (Denmark	Benzodiazepine	2 mg/kg, i.p	10 min prior to test	Saline
PACAP38	CASLO ApS (Denmark)	PAC_1_-. VPAC_1-2_ receptor agonists	2 μg/kg, s.c	1 h prior to test	Saline
Sumatriptan	GlaxoSmithKline (Denmark) via Region Hovedstaden Pharmacy (Denmark	5-HT_1B/1D/1F_ receptor agonist	0.6 mg/kg, i.p	20 min prior to PACAP38 injection	Saline

The humanized monoclonal PACAP-inhibiting antibody (anti-PACAP mAb, 5 mg/mL) together with the isogenic IgG control antibody were kindly donated by H. Lundbeck. The intraperitoneal (i.p.) administration dose of 30 mg/kg for anti-PACAP mAb was selected based on previous experiments using this drug showing effective blocking of PACAP38 induced light aversion [11]. This antibody dose is very high and corresponds to 8 nmol/mouse [11] that is a roughly 450 times concentration over exogenous PACAP38 (17 ρmol). So even though, we do not know much about the degradation of the anti-PACAP mAb in mice, we conclude that there is sufficient antibody left to block exogenous PACAP throughout the protocol of 9 days. In vitro tests showed that the anti-PACAP mAb binds both PACAP38 and the other PACAP isoform, PACAP27, with equal affinity, but is 4000-fold more selective for PACAP over VIP and does not prevent VIP induced cAMP signaling via VPAC1 and VPAC2 receptors [25].

The selective 5-HT_1B/1D/1F_ receptor agonist sumatriptan (Imigran 12 mg/mL, RegionH pharmacy) was diluted in saline to 0.06 mg/mL and administered at 0.6 mg/kg [20, 21]. The PACAP38 injection dose of 2 μg/kg was selected based on a recent in-house dose finding study that showed maximum effect at this dose [10]. PACAP38 was administered subcutaneously (s.c.) at 2 μg/kg after being diluted in saline to 0.4 μg/mL [10]. The nitric oxide (NO) donor GTN (7.89 mg/mL in 96% ethanol) was administered to mice at 10 mg/kg after being diluted in saline to 1 mg/mL [19, 20]. Levcromakalim was administered at 1 mg/kg after being dissolved in DMSO and diluted in saline with a final concentration of 2% DMSO [14, 22]. For vehicle treatment in the GTN and levcromakalim model, the same amount of ethanol and DMSO was dissolved in saline, respectively.

### Statistical analyses

We used the same statistics as in our recently published paper [10]. In short, mice groups in each cage were randomized and balanced according to 50% withdrawal thresholds measured at baseline. Treatment groups and sex were equally divided throughout the test day [26]. Group sizes were based on our previous work with these models [12, 17, 18, 20] where 12 animals per group produced sufficient power to detect intermediate and high effects [22, 27]. Cutaneous sensitivity data using Von Frey were square root transformed for improved normal distribution and data were analyzed using two-way repeated measures ANOVA. Subsequent Tukey’s *post-hoc* test was performed comparing all groups. *P* < 0.05 was considered statistically significant. Data are shown as means ± standard error of the means (SEMs). In figures, significance levels are shown as: * = *P* < 0.05, ** = *P* < 0.01, *** = *P* < 0.001, and **** = *P* < 0.0001. In the figures showing acute response, baselines are shown but were not included in the statistical analyses. Rotarod data are analyzed by Kruskal–Wallis one-way ANOVA with Dunn’s post hoc comparison. All statistical analyses and graphs were done in GraphPad Prism 9 (Graph Pad Software Inc., CA, USA).

## Results

### Anti-PACAP mAbs block PACAP38-induced hypersensitivity

**Fig. 2:**
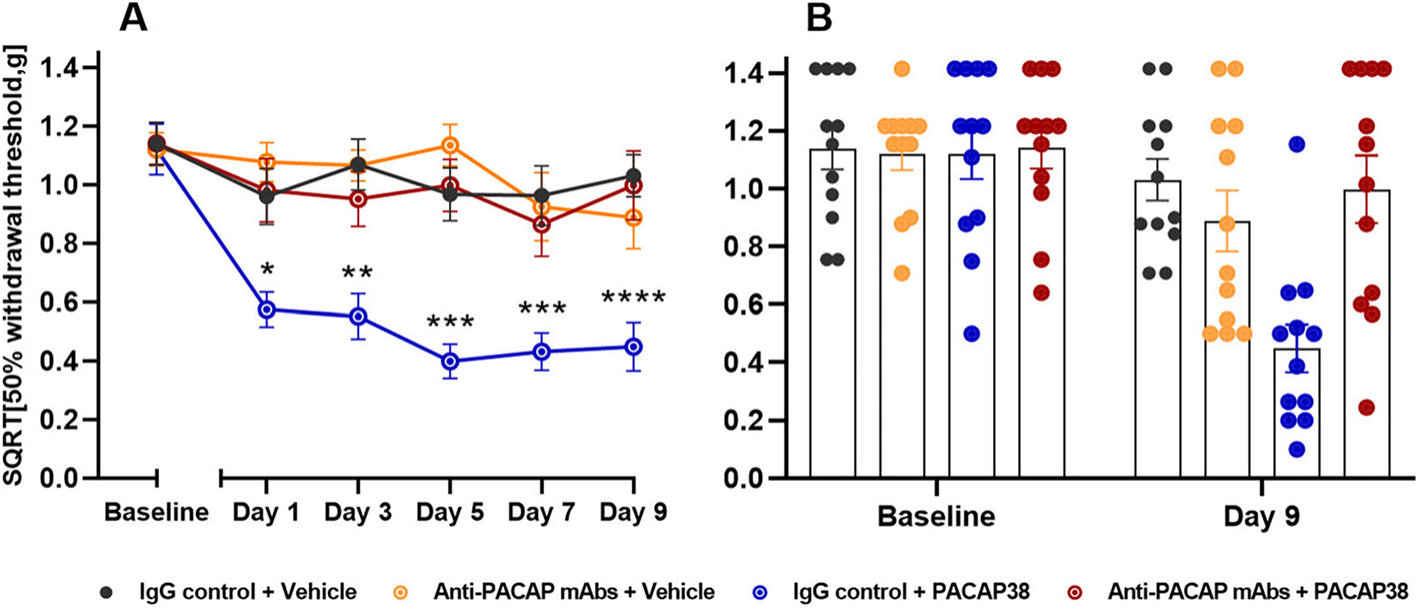
Anti-PACAP antibody block PACAP38-induced hypersensitivity. Data are shown as square root transformed (SQRT) 50% withdrawal threshold (g). **A** Responses one hour after PACAP38 (2 µg/kg, s.c.) or vehicle injection (s.c.) in wildtype mice (*n* = 12 per group) on five test days. Mice were pre-treated with anti-PACAP antibody or IgG control (30 mg/kg, i.p.) on day 0. Two-way ANOVA with Tukey’s post hoc test for multiple comparisons was used with data represented as means ± SEMs. * = *P* < 0.05, ** = *P* < 0.01, *** = *P* < 0.001, and **** = *P* < 0.0001. **B** Descriptive visualization of individual data points for baseline and day 9 of the curves on the left-hand side (Fig. 2A) with mean bars and SEMs

We tested whether anti-PACAP mAbs would be able to block PACAP38-induced hypersensitivity. A significant difference between the test group (anti-PACAP mAbs + PACAP38) and the positive control (IgG control + PACAP38) was found on all test days at the 1 h time point (*P* < 0.05 to 0.0001) (Fig. 2). The positive control group had a decrease in mean SQRT 50% withdrawal threshold from 1.12 g to 0.45 g when comparing baseline and day 9, whereas the test group had a minimal decrease from 1.14 g to 1.00 g. There was also a significant difference in basal thresholds (daily test prior to injections) on day 5,7 and 9 (*P* < 0.01 to 0.001) between the test group and the positive control group, data not shown. In addition, the test group (anti-PACAP mAbs + PACAP38) showed no difference from the negative control groups (IgG control or anti-PACAP mAbs + vehicle). Finally, the positive control group (IgG control + PACAP38) were significantly different from the negative control group (IgG control + vehicle) (*P* < 0.05 to 0.0001). The negative control group showed no significant decrease in mean SQRT 50% withdrawal threshold from 1.14 g to 1.03 g. Thus, anti-PACAP mAb blocks PACAP38-induced hypersensitivity. No adverse effects were observed following treatment with anti-PACAP mAb. All mice appeared healthy, had normal stools, weight as control mice and normal motor function.

### PACAP signaling is not involved in GTN-induced hypersensitivity

**Fig. 3:**
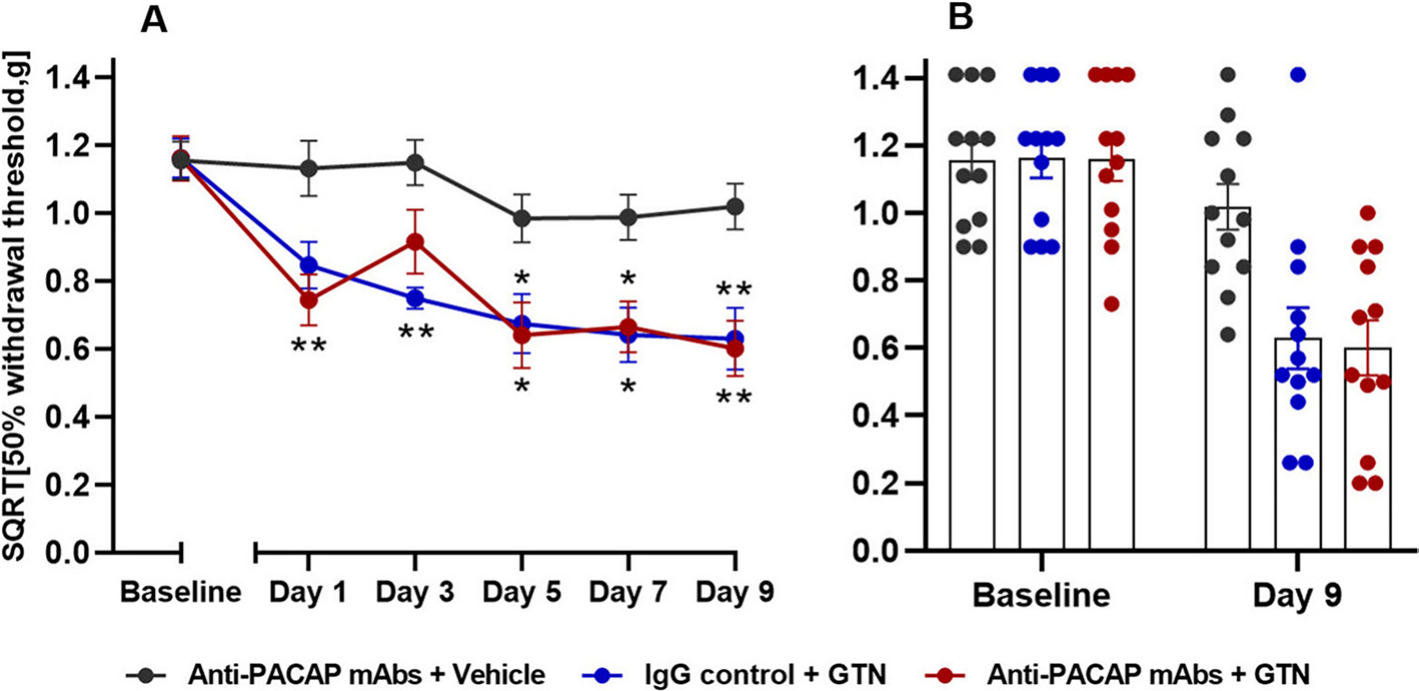
GTN signaling is independent from PACAP. Data is shown as square root transformed (SQRT)50% withdrawal threshold (g). **A** Responses two hours after GTN (10 mg/kg, i.p.) or vehicle injection (i.p.) in wildtype mice (*n* = 12 per group) on five test days. Anti-PACAP antibody or IgG control (30 mg/kg, i.p.) was given on day 0. Two-way ANOVA with Tukey’s post hoc test for multiple comparisons was used with data represented as means ± SEMs. * = *P* < 0.05, ** = *P* < 0.01, *** = *P* < 0.001, and **** = *P* < 0.0001. **B** Descriptive visualization of individual data points for baseline and day 9 of the curves on the left-hand side (Fig. 3A) with mean bars and SEMs

The effects of anti-PACAP mAb on GTN provocation was studied in wildtype mice. Both the test group (anti-PACAP mAbs + GTN) and the positive control group (IgG control + GTN) were significantly different from the negative control (anti-PACAP mAbs + vehicle) on day 5, 7 and 9 (*P* < 0.05 to 0.01) (Fig. 3). The test group had a decrease in mean SQRT 50% withdrawal threshold from 1.16 g to 0.60 g when comparing baseline and day 9, whereas the positive control group had a decrease from 1.16 g to 0.63 g. The negative control group showed only a minimal decrease in mean withdrawal threshold from 1.15 g to 1.02 g. There was also a significant difference in basal threshold on day 9 (*P* < 0.05), data not shown. No differences were seen between the test group (anti-PACAP antibody + GTN) and the positive control (IgG control + GTN). Hence, PACAP signaling is not involved in GTN-induced hypersensitivity.

### PACAP signaling is not involved in levcromakalim-induced hypersensitivity

**Fig. 4:**
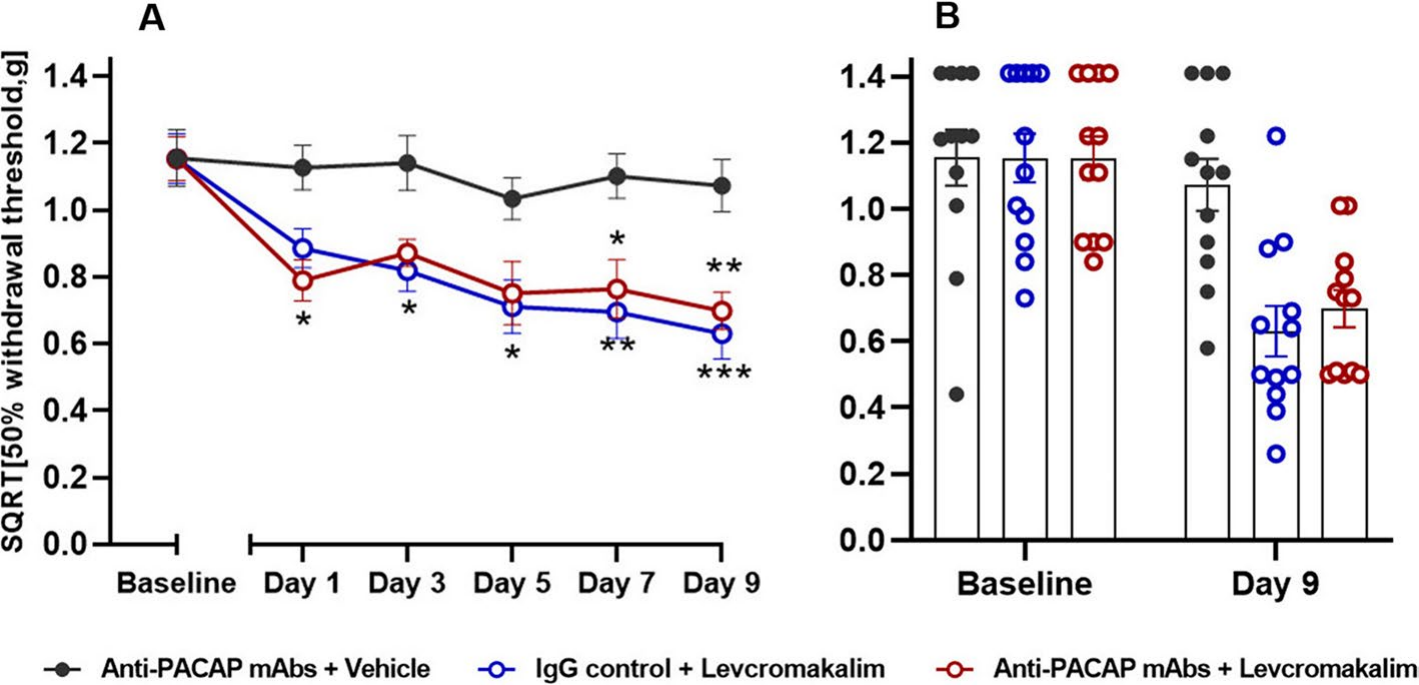
Levcromakalim signaling independent from PACAP. Data are shown as 50% withdrawal threshold (g) and was square root transformed (SQRT). **A** Acute responses two hours after levcromakalim (1 mg/kg, i.p.) or vehicle (i.p.) injection in wildtype mice (*n* = 12 per group) pre-treated with anti-PACAP antibody or IgG control (30 mg/kg, i.p.) on five test days. Two-way ANOVA with Tukey’s post hoc test for multiple comparisons was used with data represented as means ± SEMs. * = *P* < 0.05, ** = *P* < 0.01, *** = *P* < 0.001, and **** = *P* < 0.0001. **B** Descriptive visualization of individual data points for baseline and day 9 of the curves on the left-hand side (Fig. 4A) with mean bars and SEMs

The effects of anti-PACAP mAb on levcromakalim provocation was also studied in wildtype mice. Both the test group (anti-PACAP mAbs + levcromakalim) and the positive control group (IgG control + levcromakalim) were significantly different from the negative control (anti-PACAP mAb + vehicle) on day 7 and 9 (*P* < 0.05 to 0.001) (Fig. 4). The test group had a decrease in mean SQRT 50% withdrawal threshold from 1.15 g to 0.69 g when comparing baseline and day 9, whereas the positive control group had a decrease from 1.15 g to 0.63 g. For the negative control group, it only showed an insignificant decrease in mean withdrawal threshold from 1.16 g to 1.07 g. Notably, there was a significant difference in basal threshold on day 9 (*P* < 0.01 to 0.001), data not shown. No differences were seen between the test group (anti-PACAP antibody + levcromakalim) and the positive control (IgG control + levcromakalim). Therefore, PACAP signaling is not involved in levcromakalim-induced hypersensitivity.

### Sumatriptan has no effect on PACAP38-induced hypersensitivity

**Fig. 5:**
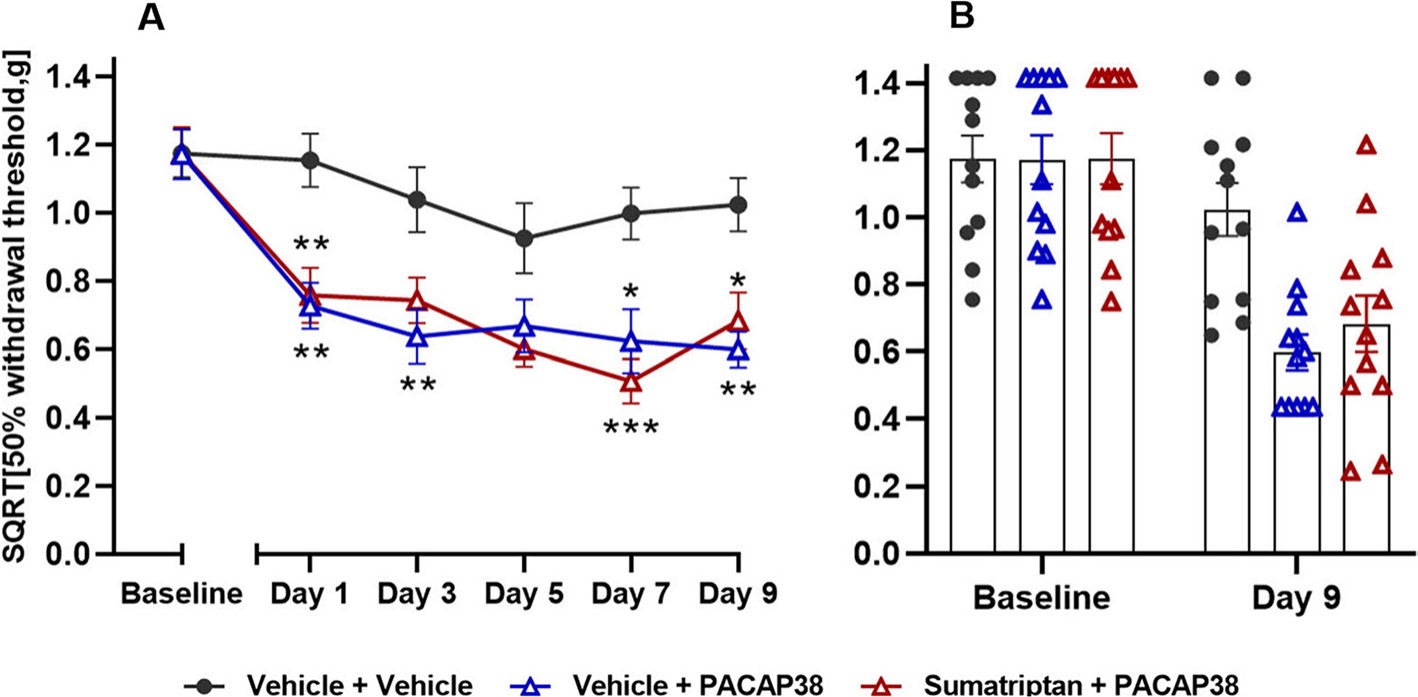
Sumatriptan had no effect on PACAP38-induced hypersensitivity. Data are shown as square root transformed (SQRT) 50% withdrawal threshold (g). **A** Responses one hour after PACAP38 (2 µg/kg, s.c.) or vehicle injection in wildtype mice (*n* = 12 per group) pre-treated with sumatriptan (0.6 mg/kg, i.p.) or vehicle (i.p.) on five test days. Two-way ANOVA with Tukey’s post hoc test for multiple comparisons was used with data represented as means ± SEMs. * = *P* < 0.05, ** = *P* < 0.01, *** = *P* < 0.001, and **** = *P* < 0.0001. **B** Descriptive visualization of individual data points for baseline and day 9 of the curves on the left-hand side (Fig. 5A) with mean bars and SEMs

The effect of sumatriptan on PACAP38 provocation was examined in wildtype mice. Both the test group (sumatriptan + PACAP38) and the positive control group (vehicle + PACAP38) were significantly different from the negative control group (vehicle + vehicle) on day 1, 7 and 9 (*P* < 0.05 to 0.001) (Fig. 5). The test group had a decrease in mean SQRT 50% withdrawal threshold from 1.17 g to 0.68 g when comparing baseline and day 9, whereas the positive control group had a decrease from 1.17 g to 0.60 g. For the negative control group, it only showed a minimal decrease in mean withdrawal threshold from 1.17 g to 1.02 g. No differences were seen between the test group (sumatriptan + PACAP38) and the positive control (vehicle + PACAP38). Hence, sumatriptan has no effect on PACAP38-induced hypersensitivity.

None of the experiments showed any evident sex differences, but the experiments were not designed and powered to detect subtle sex differences.

### Motor function was unaffected by all combinations of test substances

To assure that the von Frey tests were not biased by impaired motor coordination, this was examined in mice after injection of antibodies, PACAP38, GTN and levcromakalim using the rotarod test. We found no differences between the negative control groups (vehicle) and the tested groups (medians were 150 s in all experiments, *P* > 0.99), data not shown. As a validity of the test, we observed a significant difference between the positive control groups, which were injected with midazolam, and the tested groups (medians were 27, 27, 34 and 40, *P* < 0.0001 in all experiments), data not shown Thus, the compounds used in the experiments did not affect motor coordination. These findings were expected as these compounds have not been described to affect motor function and have not been shown to affect motor function in our previous studies [10, 22].

## Discussion

We showed that anti-PACAP mAbs blocked PACAP38 induced hypersensitivity, but not via signaling pathways involved in GTN and levcromakalim pathways. In addition, we showed that sumatriptan had no effect on PACAP38-induced hypersensitivity relevant to migraine. This is the first study to test the effect of a PACAP-inhibiting drug on GTN- and levcromakalim-induced hypersensitivity.

### PACAP and GTN

GTN is a nitric oxide (NO) donor and is an established migraine trigger used experimentally. It is blocked by anti-migraine drugs such as ibuprofen, sumatriptan [21, 28] and CGRP-inhibiting drugs [12] in the mouse model of migraine using Von Frey testing. For example, a role for peripheral CGRP in the GTN model has been clearly demonstrated in both rats and mice [12, 29–31]. The observation that PACAP blockade using mAbs did not work in our GTN model indicates a distinct pathway that does not involve peripheral PACAP.

Our finding contrasts with previous reports studying the relationship between NO, PACAP and some of its receptors. Importantly, our study can only conclude on PACAP involvement outside the CNS as the anti-PACAP mAbs is most likely peripherally restricted.

A study using a peptidomic approach identified PACAP as a mechanistic link between NO-induced chronic migraine and opioid-induced hyperalgesia in mouse models [32] and showed that PAC_1_ inhibition by a selective PAC_1_-antagonist (M65) blocked cephalic hypersensitivity induced by GTN [15]. Central neuronal PAC_1_ receptors mediated delayed activation and sensitization of trigeminocervical neurons induced by PACAP in rats [33]. Yet, another preclinical study found that peripheral blocking of PAC_1_-receptors were efficacious in an electrophysiological model [34]. Thus, both central and peripheral PAC1 receptors have been indicated as relevant dependent on the applied model. Whatsoever a phase II clinical trial of a mAb targeting the PAC_1_-receptor (AMG 301) failed to show efficacy over placebo [35].

In a mouse model of chronic migraine, repeated GTN administration increased the number of PACAP-R neurons in the trigeminal ganglion but not dorsal root ganglia [36]. In rats, GTN increased PACAP immunoreactivity within the TNC and elevated plasma PACAP concentration [37, 38]. GTN induced more photophobia, vasodilation, and trigeminal sensitization in wildtype mice compared to PACAP gene-deleted knockout mice [16]. These data differ from our present results despite sharing the GTN 10 mg/kg i.p. protocol as migraine inducer. As mentioned above, the peripheral vs central mechanisms of PACAP may be of importance here. PACAP global knockout mice possibly have altered pain transmission mechanisms in the central nervous system [39]. In contrast, anti-PACAP mAbs and PACAP38 injection may modulate dural or trigeminal nociceptors outside the brain but not cerebral receptors, as they cross the blood brain barrier very poorly [40, 41]. The central vs peripheral site of action also explains the discrepancy to the study showing increased PACAP immunoreactivity in in TNC. This is not a site reachable by the anti-PACAP mAbs applied. Interestingly, a recent study based on older data showed that injection of PACAP38 into the paraventricular nucleus of the hypothalamus increased TNC activity, which could be inhibited by an intrathecal PAC_1_ receptor antagonist [42]. Intrathecal injection of PACAP has also been suggested to induce hyperalgesia in mice [43].

### PACAP and levcromakalim

PACAP stimulates adenylyl cyclase to increase the formation of intracellular cAMP [44, 45] and there is some evidence that activation of cAMP-dependent pathway results in the opening of K_ATP_-channels [46, 47]. These findings have led to the hypothesis that modulation of nociceptive transmission by K_ATP_-channels may be a common pathway in the genesis of a migraine attack [1]. In line with this, we showed that the effect of levcromakalim—acting downstream from cAMP could not be attenuated by anti-PACAP mAbs. In our previous study, the opposite relation was studied using the same mouse model, and we showed that glibenclamide (K_ATP_-channel inhibitor) only partially inhibited PACAP38-induced hypersensitivity [10].. Taken together, existing literature and the present findings indicate that K_ATP_ channel opening induced hypersensitivity is not mediated by PACAP signaling.

### PACAP38 and sumatriptan

It is comforting when an experimental model of migraine responds to specific anti-migraine drugs, such as the triptans (5-HT_1B/1D/1F_ receptor agonist). On the other hand, demanding such effect would make it impossible to find drugs with a novel mechanism of action. Here, we showed that sumatriptan had no effect on PACAP38-induced hypersensitivity. Sumatriptan does, however, alleviate GTN-induced hypersensitivity in mice [21]. Our finding may therefore question the validity of this PACAP38 model of migraine in mice or may be interpreted as additional evidence that PACAP mediated hypersensitivity is distinct from known pathways. Our results contradict the finding of a recent randomized clinical trial showing that sumatriptan prevented PACAP38-induced migraine attacks if administered intravenously and early in 37 migraine patients [48]. Moreover, sumatriptan decreased PACAP levels measured in the external jugular vein during spontaneous migraine attacks [49], and in rodents, prolonged administration of triptan reduced brain mRNA transcription of PACAP [50]. Noteworthy, not all patients respond to sumatriptan and targeting the PACAP signaling pathways may possibly be a relevant therapeutic target in such patients.

### PACAP38 signaling pathways are distinct

PACAP38 stimulates the activity of G-protein coupled receptors for VIP, VPAC_1_ and VPAC_2_ [51]. In the trigeminovascular system, all three receptors have been documented in trigeminal[52], otic and superior cervical ganglia [53], as well as in cerebral and meningeal arteries[54]. The VPAC_1–2_ receptors also play a role in vasodilation and mast cell degranulation [55–59].

We recently showed that repeated injections of PACAP38 in wildtype mice resulted in hind paw hypersensitivity that was independent of CGRP because mice lacking *Ramp1,* a crucial part of the CGRP receptor, and mice pre-treated with mAbs against CGRP could still be sensitized by PACAP38 [10]. This differs from previous findings where CGRP antagonism was highly effective in mice sensitized by other migraine triggers e.g. GTN and levcromakalim [22]. Light aversion in mice as a surrogate for migraine-like photophobia was used to compare CGRP and PACAP38 [11]. It showed that one-third of mice did not respond to PACAP38, which was not seen with CGRP. In the same study anti-PACAP38 mAbs blocked PACAP38-induced light aversion but not CGRP-induced light aversion. Conversely, anti-CGRP mAbs could not block PACAP38-induced light aversion. Thus, our present results are in keeping with a sizable previous literature suggesting that PACAP antagonism acts independently from other migraine triggers. This further suggests that PACAP mAbs may be effective in a subgroup of migraine patients who do not respond to CGRP antagonist or sumatriptan. It also suggests that PACAP mAbs may advantageously be combined with CGRP mAbs.

### Strengths and limitations

We used of a well-validated mouse model for dissecting signaling pathways [12, 14, 17–20, 22] and the compounds used in the experiments did not affect motor coordination supporting the validity of our measurements. However, the current study uses the classical routes of administration for mice (i.p. and s.c.), which differs from intravenous infusions that is primarily used in human provocation studies [7, 60, 61]. Moreover, differences in pharmacokinetics and dynamics among species also need to be taken into consideration. Yet, the aim of the present study was to investigate underlying signaling mechanisms by inducing tactile hypersensitivity and not to reflect human dosing.

Another important methodological issue is the measurement of plantar versus periorbital sensitivity. We only measured plantar sensitivity in this study. Some researchers believe that the periorbital response is better for migraine research than plantar measurements [22]. Nevertheless, both methods are applicable in migraine research as increased cutaneous mechanical sensitivity can be induced by GTN [20, 62], levcromakalim, cilostazol [22] and PACAP38 [10] both in the plantar and periorbital areas in mice. Furthermore, as we have argued in our recently published paper [10] that both plantar and periorbital response can be inhibited by migraine-specific drugs without general analgesic effects [63, 64]. In addition, compared to the plantar area, the cutaneous sensitivity in the periorbital area has more variability and have a smaller effect window [10, 15]. This would make subtle differences more challenging to detect, and thus requires larger group sizes making the experiments less feasible [22, 62]. Likewise, other endpoints such as light sensitivity or grimacing could be relevant but they do not always report equally over time and thus also requires larger group sizes of mice due to higher variability [65].

We have not been able to replicate previous studies showing that PACAP and/or the PAC_1_ receptor is involved in mediating the effects of NO-donors. Therefore, the present study adds significantly to expand the preclinical portfolio on PACAP involvement in migraine models. Our interpretation is that if PACAP is involved in NO-induced signaling, it is not in the periphery, but centrally.

## Conclusion

Based on the findings in our mouse model of migraine using migraine-inducing compounds and anti-migraine drugs, we suggest that PACAP acts via a distinct pathway and using PACAP38 antagonism may be a novel therapeutic target of interest in a subgroup of migraine patients who do not respond to existing therapies.

## Data Availability

The datasets used or analyzed during the current study are available from the corresponding author on reasonable request.

## References

[1] Migraine AM (2020). Ropper AH, editor. N Engl J Med.

[2] Charles A, Pozo-Rosich P (2019). Targeting calcitonin gene-related peptide: a new era in migraine therapy. Lancet..

[3] Sun H, Dodick DW, Silberstein S, Goadsby PJ, Reuter U, Ashina M (2016). Safety and efficacy of AMG 334 for prevention of episodic migraine: a randomised, double-blind, placebo-controlled, phase 2 trial. Lancet Neurol.

[4] Dodick DW, Goadsby PJ, Spierings ELH, Scherer JC, Sweeney SP, Grayzel DS (2014). Safety and efficacy of LY2951742, a monoclonal antibody to calcitonin gene-related peptide, for the prevention of migraine: a phase 2, randomised, double-blind, placebo-controlled study. Lancet Neurol.

[5] Bigal ME, Edvinsson L, Rapoport AM, Lipton RB, Spierings ELH, Diener H-C (2015). Safety, tolerability, and efficacy of TEV-48125 for preventive treatment of chronic migraine: a multicentre, randomised, double-blind, placebo-controlled, phase 2b study. Lancet Neurol.

[6] Dodick DW, Goadsby PJ, Silberstein SD, Lipton RB, Olesen J, Ashina M (2014). Safety and efficacy of ALD403, an antibody to calcitonin gene-related peptide, for the prevention of frequent episodic migraine: a randomised, double-blind, placebo-controlled, exploratory phase 2 trial. Lancet Neurol.

[7] Schytz HW, Birk S, Wienecke T, Kruuse C, Olesen J, Ashina M (2009). PACAP38 induces migraine-like attacks in patients with migraine without aura. Brain.

[8] A Study With Lu AG09222 in Adults With Migraine Who Have Not Been Helped by Prior Preventive Treatments. H. Lundbeck A/S. 2022. https://clinicaltrials.gov/ct2/show/NCT05133323

[9] A Study of LY3451838 in Participants With Migraine. Eli Lilly and Company. 2022. https://clinicaltrials.gov/ct2/show/NCT04498910

[10] Ernstsen C, Christensen SL, Rasmussen RH, Nielsen BS, Jansen-Olesen I, Olesen J (2022). The PACAP pathway is independent of CGRP in mouse models of migraine: possible new drug target?. Brain.

[11] Kuburas A, Mason BN, Hing B, Wattiez AS, Reis AS, Sowers LP (2021). PACAP Induces Light Aversion in Mice by an Inheritable Mechanism Independent of CGRP. J Neurosci.

[12] Christensen SL, Petersen S, Kristensen DM, Olesen J, Munro G (2019). Targeting CGRP via receptor antagonism and antibody neutralisation in two distinct rodent models of migraine-like pain. Cephalalgia.

[13] Christensen SL, Petersen S, Sørensen DB, Olesen J, Jansen-Olesen I (2018). Cilostazol induces C-fos expression in the trigeminal nucleus caudalis and behavioural changes suggestive of headache with the migraine-like feature photophobia in female rats. Cephalalgia.

[14] Christensen SL, Munro G, Petersen S, Shabir A, Jansen-Olesen I, Kristensen DM (2020). ATP sensitive potassium (KATP) channel inhibition: A promising new drug target for migraine. Cephalalgia.

[15] Moye LS, Tipton AF, Dripps I, Sheets Z, Crombie A, Violin JD (2019). Delta opioid receptor agonists are effective for multiple types of headache disorders. Neuropharmacology.

[16] Markovics A, Kormos V, Gaszner B, Lashgarara A, Szoke E, Sandor K (2012). Pituitary adenylate cyclase-activating polypeptide plays a key role in nitroglycerol-induced trigeminovascular activation in mice. Neurobiol Dis.

[17] Ernstsen C, Christensen SL, Olesen J, Kristensen DM (2021) No additive effect of combining sumatriptan and olcegepant in the GTN mouse model of migraine. Cephalalgia 41(3):329–339 10.1177/0333102420963857 33059476

[18] Christensen SL, Ernstsen C, Olesen J, Kristensen DM (2020) No central action of CGRP antagonising drugs in the GTN mouse model of migraine. Cephalalgia 40(9):924–934 10.1177/0333102420914913 32223300

[19] Tipton AF, Tarash I, McGuire B, Charles A, Pradhan AA (2016). The effects of acute and preventive migraine therapies in a mouse model of chronic migraine. Cephalalgia.

[20] Pradhan AA, Smith ML, McGuire B, Tarash I, Evans CJ, Charles A (2014). Characterization of a novel model of chronic migraine. Pain.

[21] Bates EA, Nikai T, Brennan KC, Fu YH, Charles AC, Basbaum AI (2010). Sumatriptan alleviates nitroglycerin-induced mechanical and thermal allodynia in mice. Cephalalgia.

[22] Christensen SL, Rasmussen RH, Ernstsen C, La Cour S, David A, Chaker J (2021). CGRP-dependent signalling pathways involved in mouse models of GTN- cilostazol- and levcromakalim-induced migraine. Cephalalgia.

[23] Chaplan SR, Bach FW, Pogrel JW, Chung JM, Yaksh T (1994). Quantitative assessment of tactile allodynia evoked by unilateral ligation of the fifth and sixth lumbar nerves in the rat. J Neurosci.

[24] Christensen SL, Hansen RB, Storm MA, Olesen J, Hansen TF, Ossipov M, et al (2020) Von Frey testing revisited: Provision of an online algorithm for improved accuracy of 50% thresholds. Eur J Pain 24(4):783–790 10.1002/ejp.1528 31889375

[25] Loomis CM, Dutzar B, Ojala EW, Hendrix L, Karasek C, Scalley-Kim M (2019). Pharmacologic Characterization of ALD1910, a Potent Humanized Monoclonal Antibody against the Pituitary Adenylate Cyclase-Activating Peptide. J Pharmacol Exp Ther.

[26] Minett MS, Eijkelkamp N, Wood JN (2014). Significant determinants of mouse pain behaviour. PLoS One.

[27] Potvin PJ, Schutz RW (2000). Statistical power for the two-factor repeated measures ANOVA. Behav Res Methods Instrum Comput.

[28] Olesen J, Jansen-Olesen I (2012). Towards a reliable animal model of migraine. Cephalalgia.

[29] Greco R, Demartini C, Zanaboni AM, Tassorelli C (2018). Chronic and intermittent administration of systemic nitroglycerin in the rat induces an increase in the gene expression of CGRP in central areas: potential contribution to pain processing. J Headache Pain.

[30] Capuano A, Greco MC, Navarra P, Tringali G (2014). Correlation between algogenic effects of calcitonin-gene-related peptide (CGRP) and activation of trigeminal vascular system, in an in vivo experimental model of nitroglycerin-induced sensitization. Eur J Pharmacol.

[31] Greco R, Mangione AS, Siani F, Blandini F, Vairetti M, Nappi G (2014). Effects of CGRP receptor antagonism in nitroglycerin-induced hyperalgesia. Cephalalgia.

[32] Anapindi KDB, Yang N, Romanova EV, Rubakhin SS, Tipton A, Dripps I (2019). PACAP and other neuropeptide targets link chronic migraine and opioid-induced hyperalgesia in mouse models. Mol Cell Proteomics.

[33] Akerman S, Goadsby PJ (2015). Neuronal PAC1 receptors mediate delayed activation and sensitization of trigeminocervical neurons: Relevance to migraine. Sci Transl Med.

[34] Hoffmann J, Miller S, Martins-Oliveira M, Akerman S, Supronsinchai W, Sun H (2020). PAC1 receptor blockade reduces central nociceptive activity: new approach for primary headache?. Pain.

[35] Ashina M, Doležil D, Bonner JH, Zhou L, Klatt J, Picard H (2021). A phase 2, randomized, double-blind, placebo-controlled trial of AMG 301, a pituitary adenylate cyclase-activating polypeptide PAC1 receptor monoclonal antibody for migraine prevention. Cephalalgia.

[36] Guo Z, Czerpaniak K, Zhang J, Cao YQ (2021). Increase in trigeminal ganglion neurons that respond to both calcitonin gene-related peptide and pituitary adenylate cyclase-activating polypeptide in mouse models of chronic migraine and posttraumatic headache. Pain.

[37] Farajdokht F, Babri S, Karimi P, Alipour MR, Bughchechi R, Mohaddes G (2017). Chronic ghrelin treatment reduced photophobia and anxiety-like behaviors in nitroglycerin- induced migraine: role of pituitary adenylate cyclase-activating polypeptide. Eur J Neurosci.

[38] Farajdokht F, Mohaddes G, Shanehbandi D, Karimi P, Babri S (2018). Ghrelin attenuated hyperalgesia induced by chronic nitroglycerin: CGRP and TRPV1 as targets for migraine management. Cephalalgia.

[39] Sándor K, Kormos V, Botz B, Imreh A, Bölcskei K, Gaszner B (2010). Impaired nocifensive behaviours and mechanical hyperalgesia, but enhanced thermal allodynia in pituitary adenylate cyclase-activating polypeptide deficient mice. Neuropeptides.

[40] Dogrukol-Ak D, Tore F, Tuncel N (2004). Passage of VIP/PACAP/secretin family across the blood-brain barrier: therapeutic effects. Curr Pharm Des.

[41] Schytz HW, Olesen J, Ashina M (2010). The PACAP receptor: a novel target for migraine treatment. Neurotherapeutics.

[42] Robert C, Bourgeais L, Arreto C-D, Condes-Lara M, Noseda R, Jay T (2013). Paraventricular hypothalamic regulation of trigeminovascular mechanisms involved in headaches. J Neurosci.

[43] Narita M, Dun SL, Dun NJ, Tseng LF (1996). Hyperalgesia induced by pituitary adenylate cyclase-activating polypeptide in the mouse spinal cord. Eur J Pharmacol.

[44] Miyata A, Arimura A, Dahl RR, Minamino N, Uehara A, Jiang L (1989). Isolation of a novel 38 residue-hypothalamic polypeptide which stimulates adenylate cyclase in pituitary cells. Biochem Biophys Res Commun.

[45] Spongier D, Waeber C, Pantaloni C, Holsboer F, Bockaert J, Seeburgt PH (1993). Differential signal transduction by five splice variants of the PACAP receptor. Nature.

[46] Armstead WM (1996). Role of ATP-sensitive K+ channels in cGMP-mediated pial artery vasodilation. Am J Physiol.

[47] Wellman GC, Quayle JM, Standen NB (1998). ATP-sensitive K+ channel activation by calcitonin gene-related peptide and protein kinase A in pig coronary arterial smooth muscle. J Physiol.

[48] Wienholtz NKF, Christensen CE, Zhang DG, Coskun H, Ghanizada H, Al-Karagholi MAM (2021). Early treatment with sumatriptan prevents PACAP38-induced migraine: A randomised clinical trial. Cephalalgia.

[49] Zagami AS, Edvinsson L, Goadsby PJ (2014). Pituitary adenylate cyclase activating polypeptide and migraine. Ann Clin Transl Neurol.

[50] Muzzi M, Buonvicino D, De Cesaris F, Chiarugi A (2017). Acute and chronic triptan exposure neither alters rodent cerebral blood flow nor worsens ischemic brain injury. Neuroscience.

[51] Dickson L, Finlayson K (2009). VPAC and PAC receptors: From ligands to function. Pharmacol Ther.

[52] Hou M, Uddman R, Tajti J, Edvinsson L (2003). Nociceptin immunoreactivity and receptor mRNA in the human trigeminal ganglion. Brain Res.

[53] Knutsson M, Edvinsson L (2002). Distribution of mRNA for VIP and PACAP receptors in human cerebral arteries and cranial ganglia. NeuroReport.

[54] Chan KY, Baun M, De Vries R, Van Den Bogaerdt AJ, Dirven CMF, Danser AHJ (2011). Pharmacological characterization of VIP and PACAP receptors in the human meningeal and coronary artery. Cephalalgia.

[55] Boni L, Ploug K, Olesen J, Jansen-Olesen I, Gupta S (2009). The in vivo effect of VIP, PACAP-38 and PACAP-27 and mRNA expression of their receptors in rat middle meningeal artery. Cephalalgia.

[56] Kulka M, Sheen CH, Tancowny BP, Grammer LC, Schleimer RP (2008). Neuropeptides activate human mast cell degranulation and chemokine production. Immunology.

[57] Tajti J, Tuka B, Botz B, Helyes Z, Vecsei L (2015). Role of pituitary adenylate cyclase-activating polypeptide in nociception and migraine. CNS Neurol Disord Drug Targets.

[58] Fahrenkrug J, Hannibal J, Tams J, Georg B (2000). Immunohistochemical localization of the VIP1 receptor (VPAC1R) in rat cerebral blood vessels: Relation to PACAP and VIP containing nerves. J Cereb Blood Flow Metab.

[59] Schmidt-Choudhury A, Furuta GT, Galli SJ, Schmidt WE, Wershil BK (1999). Mast cells contribute to PACAP-induced dermal oedema in mice. Regul Pept.

[60] Lassen LH, Haderslev PA, Jacobsen VB, Iversen HK, Sperling B, Olesen J (2002). CGRP may play a causative role in migraine. Cephalalgia.

[61] Thomsen LL, Kruuse C, Iversen HK, Olesen J (1994). A nitric oxide donor (nitroglycerin) triggers genuine migraine attacks. Eur J Neurol.

[62] Ben Aissa M, Tipton AF, Bertels Z, Gandhi R, Moye LS, Novack M (2018). Soluble guanylyl cyclase is a critical regulator of migraine-associated pain. Cephalalgia.

[63] Brennum J, Brinck T, Schriver L, Wanscher B, SoelbergSørensen P, Tfelt-Hansen P (1996). Sumatriptan has no clinically relevant effect in the treatment of episodic tension-type headache. Eur J Neurol.

[64] Moreno-Ajona D, Pérez-Rodríguez A, Goadsby PJ (2020). Gepants, calcitonin-gene-related peptide receptor antagonists. Curr Opin Neurol.

[65] Christensen SLT, Petersen S, Sørensen DB, Olesen J, Jansen-Olesen I (2016). Infusion of low dose glyceryl trinitrate has no consistent effect on burrowing behavior, running wheel activity and light sensitivity in female rats. J Pharmacol Toxicol Methods.

