# Correction: PACAP signaling is not involved in GTN- and levcromakalim-induced hypersensitivity in mouse models of migraine

**DOI:** 10.1186/s10194-023-01606-0

**Published:** 2023-06-19

**Authors:** Song Guo, Charlotte Ernstsen, Anders Hay-Schmidt, David Møbjerg Kristensen, Messoud Ashina, Jes Olesen, Sarah Louise Christensen

**Affiliations:** 1 Department of Neurology, Danish Headache Center, Research Institute, Copenhagen University Hospital-Rigshospitalet Glostrup, Nordstjernevej 42, Glostrup, 2600 Copenhagen, Denmark; 2 Department of Odontology, Faculty of Health, Panum Institute, University of Copenhagen, Copenhagen, Denmark; 3 Department of Science and Environment, Roskilde University, Universitetsvej 1, Rosklide, Denmark; 4 Univ Rennes, INSERM, EHESP, Irset (Institut de Recherche en Santé, Environnement Et Travail) - UMR_S 1085, Rennes, France; 5 Department of Neurology, Danish Headache Center, Human Migraine Research Unit, Copenhagen University Hospital Rigshospitalet-Glostrup, Copenhagen, Denmark

**Correction: J Headache Pain 23, 155 (2022)**

**https://doi.org/10.1186/s10194-022-01523-8**

In this article [1], the author name of David Møbjerg Kristensen was unintentionally omitted.

The author list has been updated and the original article has been corrected.

The authors would like to apologise for any inconvenience caused.
